# Plasmid-Mediated Quinolone Resistance in *Shigella flexneri* Isolated From Macaques

**DOI:** 10.3389/fmicb.2018.00311

**Published:** 2018-03-05

**Authors:** Anthony J. Mannion, Heather R. Martin, Zeli Shen, Ellen M. Buckley, JoAnn L. Dzink-Fox, Alexis Garcia, Robert P. Marini, Mary M. Patterson, James G. Fox

**Affiliations:** Division of Comparative Medicine, Massachusetts Institute of Technology, Cambridge, MA, United States

**Keywords:** plasmid-mediated quinolone resistance, enrofloxacin resistance, multi-drug antibiotic resistance, *Shigella flexneri*, non-human primates, zoonotic risk

## Abstract

Non-human primates (NHPs) for biomedical research are commonly infected with *Shigella* spp. that can cause acute dysentery or chronic episodic diarrhea. These animals are often prophylactically and clinically treated with quinolone antibiotics to eradicate these possible infections. However, chromosomally- and plasmid-mediated antibiotic resistance has become an emerging concern for species in the family *Enterobacteriaceae*. In this study, five individual isolates of multi-drug resistant *Shigella flexneri* were isolated from the feces of three macaques. Antibiotic susceptibility testing confirmed resistance or decreased susceptibility to ampicillin, amoxicillin-clavulanic acid, cephalosporins, gentamicin, tetracycline, ciprofloxacin, enrofloxacin, levofloxacin, and nalidixic acid. *S. flexneri* isolates were susceptible to trimethoprim-sulfamethoxazole, and this drug was used to eradicate infection in two of the macaques. Plasmid DNA from all isolates was positive for the plasmid-encoded quinolone resistance gene *qnrS*, but not *qnrA* and *qnrB*. Conjugation and transformation of plasmid DNA from several *S. flexneri* isolates into antibiotic-susceptible *Escherichia coli* strains conferred the recipients with resistance or decreased susceptibility to quinolones and beta-lactams. Genome sequencing of two representative *S. flexneri* isolates identified the *qnrS* gene on a plasmid-like contig. These contigs showed >99% homology to plasmid sequences previously characterized from quinolone-resistant *Shigella flexneri* 2a and *Salmonella enterica* strains. Other antibiotic resistance genes and virulence factor genes were also identified in chromosome and plasmid sequences in these genomes. The findings from this study indicate macaques harbor pathogenic *S. flexneri* strains with chromosomally- and plasmid-encoded antibiotic resistance genes. To our knowledge, this is the first report of plasmid-mediated quinolone resistance in *S. flexneri* isolated from NHPs and warrants isolation and antibiotic testing of enteric pathogens before treating macaques with quinolones prophylactically or therapeutically.

## Introduction

*Shigella* spp., particularly *Shigella flexneri*, are common enteric pathogens isolated from non-human primates (NHPs), including macaques, that can cause acute dysentery and are associated with chronic episodic diarrhea. In addition to the lower bowel, *Shigella* spp. can also colonize the inflamed gingival mucosa (McClure et al., 1976; Armitage et al., 1982). Clinically, animals may not appear ill, but *Shigella* spp. can be cultured from normally formed, but blood-streaked feces. These animals represent *Shigella* spp. reservoirs capable of transmitting the pathogen to other colony-maintained NHPs and pose important zoonotic risks to research, veterinary, and zoo personnel (Fox, 1975; Tribe and Fleming, 1983; Kennedy et al., 1993).

Because of the high incidence of diarrhea in newly imported NHPs, antibiotics are often given prophylactically to reduce its clinical severity. Enrofloxacin, a fluoroquinolone antibiotic, is commonly prescribed because of its efficacy in treating gram-negative pathogens and its convenience of a once-daily parenteral treatment. In addition, enrofloxacin has also been used to successfully eradicate *Shigella* spp. from a variety of NHP species housed in a laboratory and zoological setting (Line et al., 1992; Banish et al., 1993). However, widespread and prophylactic use of enrofloxacin and other antibiotics act as selective pressures for the emergence of antibiotic resistant organisms.

Quinolone resistance is typically chromosomally-mediated; however, an increasing finding among species in the family *Enterobacteriaceae* is plasmid-mediated quinolone resistance due to *qnr* genes. While several homologs of *qnr* genes have been discovered, the best characterized are the *qnrA, qnrB*, and *qnrS* genes (Strahilevitz et al., 2009; Redgrave et al., 2014). These genes encode a ~218 amino acid protein that protects bacterial DNA gyrase and topoisomerase from quinolone and fluoroquinolone inhibition, thus resulting in low-level quinolone resistance (Tran and Jacoby, 2002; Tran et al., 2005a,b; Redgrave et al., 2014). Since first being reported in 1998 from a clinical isolate of *Klebsiella pneumoniae* (Martinez-Martinez et al., 1998), plasmid-mediated quinolone resistance has been reported in other *Enterobacteriaceae* species throughout the United States (Wang et al., 2004) and elsewhere in the world (Hata et al., 2005; Mammeri et al., 2005; Strahilevitz et al., 2009; Karczmarczyk et al., 2012).

Plasmids encoding *qnr* often harbor other antibiotic resistance genes and can be transferred to other bacterial species by conjugation (Martinez-Martinez et al., 1998). For example, the ~47 kb, conjugative plasmid pAH0376 was discovered in *S. flexneri* 2b isolates that caused a food poisoning outbreak and harbors both a TEM-1 β-lactamase gene and *qnrS* gene (Hata et al., 2005). Thus, plasmid-mediated quinolone resistance represents a significant public health concern because they act as vectors that propagate multi-drug antibiotic resistant pathogens.

Given the frequent use of quinolones and potential for zoonotic transmission, the purpose of this report is to describe the isolation of plasmid-mediated multiple-antibiotic resistant *S. flexneri* strains recovered from three colony-maintained macaques, and the successful eradication of these drug-resistant *S. flexneri* isolates.

## Materials and methods

### Case history

Macaques (*Macaca mulatta*), obtained from a United States-based vendor in 2005, underwent physical examination and routine diagnostic evaluations during quarantine and thereafter at quarterly intervals. Macaques were routinely pair-housed and maintained in an AAALAC International-accredited animal facility. They were fed specified amounts of primate chow (Purina Lab Diet® 5038, St. Louis, MO) twice daily and provided water *ad libitum*. Housing conditions were maintained between 20 and 22°C, 30–70% humidity, 10–15 non-recirculated air changes per hour, and a light cycle of 12 h light, 12 h of dark per day.

During a quarterly physical and diagnostic evaluation, macaque 05-18 presented with fresh blood in its feces. Fecal samples were collected directly from the rectum and submitted for culture and antibiotic sensitivity. Shortly after the initial fecal sample was collected, macaque 05-18 and its cagemate underwent antibiotic treatment with enrofloxacin (5 mg/kg IM every 24 h for 10 days) following research-related surgical procedures. *S. flexneri* variant Y (strain 06-2384) was cultured from the original submission and found sensitive to trimethoprim-sulfamethoxazole, but resistant to ampicillin, amoxicillin-clavulanic acid, cephalothin, gentamicin, and enrofloxacin using Kirby-Bauer antibiotic testing. The two clinically normal monkeys were placed in quarantine together and a set of rectal and gingival swabs were submitted for culture. The second culture sample, as well as fecal samples collected twice more at 2-week intervals, were negative for *Shigella* spp. Following treatment with enrofloxacin, multiple follow-up fecal cultures were negative for *Shigella* spp.

Fecal samples were collected from all macaques (*N* = 24) and submitted for culture and antibiotic susceptibility testing during the same time interval the shigella*-*positive macaque, 05-18, was found. Two additional macaques, 05-15 and 05-16, housed in the adjacent cage to macaque 05-18, had fecal cultures positive for *S. flexneri* type 5b, designated 06-2835 and 06-2836, respectively. This serotype also showed multiple-antibiotic resistance, with intermediate sensitivity to enrofloxacin and sensitivity to trimethoprim-sulfamethoxazole. The second pair was moved to quarantine and treated with enrofloxacin (5 mg/kg IM every 24 h for 10 days). However, follow-up fecal cultures from the cage pan were still positive for *S. flexneri* type 5b, designated strain 06-3102. Subsequently, the pair was treated with trimethoprim-sulfadiazine (30 mg/kg SQ every 24 h for 10 days); all subsequent cultures were negative for *Shigella* spp.

### *Shigella* isolation

Cage pan feces and rectal swabs collected from macaques were swabbed onto XLD (xylose, lysine, deoxycholate) and HE (hektoen-enteric) agar media, and the samples were then placed into selenite broth (Remel, Inc., Lenexa, KS.). All media were incubated at 37°C for 18 to 24 h, after which the selenite broth was sub-cultured onto the same media and re-incubated. Isolates that were transparent on the XLD and HE agars were sub-cultured on BAP (Trypticase Soy Agar with 5% Sheep Blood; Remel) and identified as *Shigella* spp. by API 20E (bioMerieux, Inc., Hazelwood, MO). Isolates were submitted to the Texas Department of State Health Services lab for serotyping.

### Antimicrobial agents

Antibiotics used for broth microdilution assays (ampicillin, azithromycin, cefazolin, cefoxitin, ceftazidime, ceftriaxone, cephalothin, ciprofloxacin, clarithromycin, enrofloxacin, gentamicin, imipenem, levofloxacin, nalidixic acid, and tetracycline powders) were purchased from the Sigma Chemical Company (St. Louis, MO). Amoxicillin-clavulanic acid and trimethoprim-sulfamethoxazole were obtained as E-test strips from bioMerieux (Durham, NC.). Disks of ampicillin (10 μg), cephalothin (30 μg), enrofloxacin (5 μg), nalidixic acid (30 μg), tetracycline (30 μg), amoxicillin-clavulanic acid (30 μg), and trimethoprim-sulfamethoxazole (25 μg) were obtained from Remel (Lenexa, KS.).

### Antimicrobial susceptibility testing

Antimicrobial susceptibility tests were initially performed by disk diffusion in accordance with the Clinical Laboratory Standards Institute (2010). In addition, to determine the level of sensitivity, antibiotic mean inhibitory concentrations (MICs) were determined by the broth microdilution method using 2-fold serial dilutions, as recommended by CLSI (Clinical Laboratory Standards Institute, 2012, 2014). MIC is defined as the lowest concentration of an antimicrobial agent that inhibits visible growth. *Escherichia coli* (ATCC25922) was used as the standard reference strain. Experiments were performed in triplicate from two or more independent experiments.

### DNA extraction

Whole DNA (includes both chromosomal and plasmid DNA) from the five *S. flexneri* isolates was extracted using the High Pure PCR Template Preparation Kit (Roche Molecular Biochemicals, Indianapolis, IN). Plasmid DNA was extracted using the QIAprep Spin Miniprep Kit (QIAGEN Inc. Valencia, CA).

### Screening of *qnr* antimicrobial-resistance genes

Screening for the *qnrA, qnrB*, and *qnrS* genes was performed on whole and plasmid DNA using the primers and PCR conditions described previously (Robicsek et al., 2006). PCR products were concentrated and purified with the QIAquick PCR purification kit (Qiagen, Valencia, CA) for sequencing with an ABI Prism cycle sequencing kit (BigDye Terminator cycle sequencing kit) on an ABI 3100 genetic analyzer (Applied Biosystems, Foster City, CA). Sequences were analyzed by BLAST hosted by the National Center for Biotechnology Information (NCBI) to identify homologous genes and calculate percent identity.

### Conjugation and transformation assays

Conjugation experiments were performed using the *S. flexneri* isolates as donors and *E. coli* J53 Az^r^ as the recipients (Wang et al., 2004). Cultures of donor and recipient cells were grown in 3 mL of fresh Luria Bertani (LB) broth (BBL®) at 37°C with shaking for 3 h. The conjugation assay was then performed in 3 mL of fresh LB broth using the donor and recipient cells in logarithmic phase (0.5 mL each) and incubated at 37°C for 3 h without shaking. Transconjugants were selected on trypticase soy agar plates (TSA; BBL®) containing sodium azide (100 μg/mL: Sigma-Aldrich Corporation, St. Louis, Mo.) for counter-selection, and ampicillin (50 μg/mL; Sigma-Aldrich Corp.) for selection of plasmid-encoded resistance (Fox, 1975; Mammeri et al., 2005; Cattoir et al., 2007). Colonies were also plated on TSA plates containing sodium azide (100 μg/mL) and nalidixic acid (16 μg/mL). The transconjugants were plated onto MacConkey agar plates, and their identities were reconfirmed as *E. coli* using with API 20E.

For transformation experiments, 5 μL of plasmid DNA from four donor strains was heat shocked at 42°C for 30 s into chemically component *E. coli* Top10 cells. The cells were then incubated in 250 μL of S.O.C. (super optimal broth with catabolic repressor) for 1 h at 37°C, followed by overnight growth on LB plates supplemented with ampicillin (50 μg/mL; Sigma-Aldrich Corp.) for selection of antibiotic-resistant transformants. Colonies were screened by PCR for the *qnrS* gene. MICs for the donors, recipient, transconjugants, and transformants were measured as described above.

### Whole genome sequencing of representative *S. flexneri* isolates

Whole DNA was extracted from two representative *S. flexneri* isolates, 06-2384 and 06-3102, using the High Pure PCR Template Preparation Kit (Roche Molecular Biochemicals, Indianapolis, IN). These isolates were chosen in order to represent all infected animals (06-2384 cultured from monkey 05-18; 06-3102 cultured from co-housed monkeys 05-15 and 05-16) as well as the potential genetic diversity of both serotypes (variant Y and 5b, corresponding to 06-2384 and 06-3102, respectively). DNA was then prepared into libraries using the SMRTbell Template Prep Kit 1.0 and the DNA/Polymerase Binding Kit P6 v2 (Pacific Biosciences, Menlo Park, CA). DNA libraries were size-selected for fragments ≥~5kb and then were sequenced on a single SMRT cell per genome using a Pacific Biosciences RSII sequencer at the University of Massachusetts Deep Sequencing Core Facility (Worchester, MA). Sequencing reads were quality filtered and trimmed for *de novo* assembly using the Hierarchical Genome Assembly Process (HGAP 3.0) workflow hosted on the SMRT Portal 2.3. Resulting contigs were annotated using RASTtk (Aziz et al., 2008) hosted by Pathosystems Resource Integration Center (PATRIC) (Wattam et al., 2014, 2017). VirulenceFinder 1.5 (Joensen et al., 2014), ResFinder 2.1 (Zankari et al., 2012), and PlasmidFinder 1.3 (Carattoli et al., 2014) were used to identify virulence factors, antibiotic resistance genes, and plasmids, respectively. *In silico* multi-locus sequencing type (MLST) was determined using MLST-1.6 (Larsen et al., 2012) against the *E. coli* #1 allelic profile database. Average nucleotide identity (ANI) was calculated with OrthoANIu (Lee et al., 2016), and digital DNA-DNA hybridization (dDDH) was calculated with Genome-to-Genome Distance Calculator (GGDC) 2.1 (Auch et al., 2010). Graphical circular maps of chromosome and plasmid sequences were generated using the CGView Server (Grant and Stothard, 2008).

## Results

### *Shigella* spp. identification

*S. flexneri* isolates were identified using selective agar and API 20E strips and were also serotyped by the Texas Department of State Health Services. *S. flexneri* variant Y strain 06-2384 was isolated in the index case (*i.e.*, monkey 05-18). *S. flexneri* type 5b strains 06-2835 R1 and 06-2835 R2, which had different colony morphologies and API codes, were isolated from monkey 05-15. Strain 06-2836 was cultured from monkey 05-16. Following unsuccessful enrofloxacin treatment of paired monkeys 05-15 and 05-16, feces were collected from the cage pan and strain 06-3102 was cultured. These *S. flexneri* isolates were then analyzed for antibiotic susceptibility, the presence of *qnr* genes by PCR, and the ability to transfer plasmid-encoded antibiotic resistance genes to *E. coli* recipients by conjugation and transformation.

### *qnrS1* gene detected in *S. flexneri* strains isolated from macaques

PCR detected *qnrS*, but not *qnrA* or *qnrB*, in both whole and plasmid DNA from all five *S. flexneri* isolates (Supplementary Figure 1). BLAST analysis of three representative *qnrS* PCR product sequences against the nr/nt database determined they were 100% identical to the plasmid-encoded *qnrS1* genes found in other *Enterobacteriaceae* species, including pAH0376 from *Shigella flexneri* 2b and pVQS1 from *Salmonella enterica*, indicating these novel *S. flexneri* strains harbor the *qnrS1* variant.

### Antimicrobial resistance transferrable by plasmid conjugation and transformation

Antimicrobial susceptibility testing of four *S. flexneri* isolates by microdilution indicated multi-drug resistance to ampicillin, cephalothin, ceftazidime, ceftriaxone, cefazolin, gentamicin (except strain 06-2835), nalidixic acid, and tetracycline and decreased susceptibility to amoxicillin-clavulanic acid, ciprofloxacin, enrofloxacin, and levofloxacin compared to the *E. coli* control strain ATCC25922 (Table 1). The *S. flexneri* isolates were in general susceptible to azithromycin, imipenem, cefoxitin, trimethoprim-sulfamethoxazole (Table 1).

**Table 1:** MIC (μg/mL)^†^.

**Antibiotic class**		***Shigella flexneri*** **donor (origin)**				***E. coli*** **J53 transconjugants**					***E. coli*** **Top10 transformants**					**Control** ***E. coli***	
		**06-2384**	**06-2835**	**06-2836**	**06-3102**	**Naïve** **strain**	**06-2384**	**06-2835**	**06-2836**	**06-3102**	**Naïve** **strain**	**06-2384**	**06-2835**	**06-2836**	**06-3102**	***E. coli* ATCC 25922**	**Breakpoint resistance**
Macrolide	Azithromycin	1–2	1–2	2	2	8	8	8	8	8-16	4–8	4	4–8	8	4	4–8	N/A
	Clarithromycin	64	32–64	32–64	32–64	64–128	128	128	64	64–128	64–128	128	32–64	>128	64–128	64–128	N/A
Cephalosporin	Cephalothin	32–64	>128	>128	>128	16	32–64	32–64	>128	64	32	32	64–128	>128	32–64	32	≥32
	Ceftazidime	16	128	128	128	N/T	N/T	N/T	N/T	N/T	N/T	N/T	N/T	N/T	N/T	0.125	≥16
	Ceftriaxone	8	>128	>128	>128	N/T	N/T	N/T	N/T	N/T	N/T	N/T	N/T	N/T	N/T	0.06	≥4
	Cefazolin	32	>128	>128	>128	N/T	N/T	N/T	N/T	N/T	N/T	N/T	N/T	N/T	N/T	2	≥8
	Cefoxitin	4	4	4	4	N/T	N/T	N/T	N/T	N/T	N/T	N/T	N/T	N/T	N/T	4	≥32
Aminoglycoside	Gentamicin	>128	0.5–1	>128	>128	2	1–2	2	2	2–4	0.5–1	1	0.5–1	1–2	1–2	1–2	≥16
Tetracycline	Tetracycline	128	128	128	128	2	2	2	2	2–4	2–4	2	2–4	2	2–4	1–2	≥16
Quinolone	Nalidixic Acid	32	32–64	32–64	32	4–8	32	16	16	16	1–2	4–16	8–32	8	4–8	2–4	≥32
	Ciprofloxacin	1–2	1–2	1–2	1–2	0.015	0.5–1	0.5–1	0.5	0.5–1	<0.015	0.25–0.5	0.25–0.5	0.5	0.25–0.5	<0.015	≥4
	Enrofloxacin	1–4	1–4	1–4	1–4	0.03–0.06	1–2	0.125–2	1	1–2	<0.015	0.125–1	0.25–2	0.125–0.5	0.125–0.5	<0.015	≥4
	Levofloxacin	0.5–1	0.5–2	0.5–1	1–2	0.03–0.06	0.5–1	0.25–0.5	0.5–1	0.5–1	<0.015	0.125–0.25	0.25–0.5	0.25	0.125–0.25	<0.015	≥8
Beta-Lactam	Ampicillin	>256	>256	>256	>256	4	>256	>256	>256	>256	4–8	>256	>256	>256	>256	2–4	≥32
	Imipenem	0.03	0.03	0.03	0.03	N/T	N/T	N/T	N/T	N/T	N/T	N/T	N/T	N/T	N/T	0.03	≥4
	Amoxicillin-Clavulanic Acid	16	12	12–16	12	4	6–8	8–12	12	12	4	16	16	16	16	4–6	≥32
Sulfonamide	Trimethoprim-Sulfamethoxazole	0.064	0.064	0.064–0.094	0.064	0.064	0.19	0.064	0.064	0.064	0.032	0.032	0.047	0.047	0.032	0.19	≥4

† *N/A, not available; N/T, not tested*.

**Figure 1:**
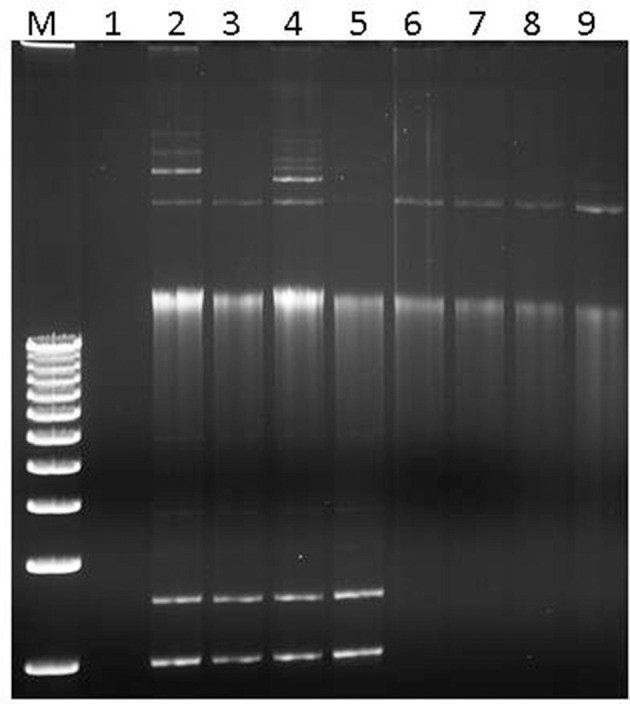
Plasmid separation profiles of the donor *S. flexneri* isolates, naïve *E. coli* recipient strain, and *E. coli* transformants. Lane 1: naïve *E. coli* Top10 recipient. Lanes 2–5: *S. flexneri* isolates 06-3102, 06-2384, 06-2835, 06-2836. Lanes 6–9: *E. coli* transformants t-06-3102, t-06-2384, t-06-2835, t-06-2836. Similar results were obtained for the transconjugants (not shown).

Plasmid DNA from these *S. flexneri* isolates were successfully conjugated and transformed into the *E. coli* recipient strains J53 Az^r^ and Top10, respectively. Plasmid DNA isolated from all the transconjugants and transformants were PCR-positive for the *qnrS* gene (Supplementary Figure 2). Likewise, plasmid profiles were similar between the donor *S. flexneri* isolates and the *E. coli* transconjugants and transformants, indicating successful transfer of plasmid DNA to the recipients (Figure 1). The *S. flexneri* isolates have several bands in the plasmid profile, indicating they may harbor multiple different plasmids.

Compared to the naïve *E. coli* recipient strains J53 Az^r^ and Top10, the transconjugants and transformants demonstrated increased resistance to all quinolone antibiotics tested (Table 1). The MIC of the fluoroquinolones was 8- to 32-fold higher in the transconjugants and transformants, while nalidixic acid showed a 2- to 8-fold increase versus their naïve *E. coli* recipient strains (Table 1). The transconjugants and transformants also had increased resistance to amoxicillin-clavulanic acid as compared to the naïve *E. coli* recipient strains. All transconjugants and transformants were highly resistant to ampicillin and several cephalosporins, characteristic of beta-lactamase activity. The transconjugants and transformants demonstrated similar susceptibility to azithromycin, clarithromycin, gentamicin, tetracycline, and trimethoprim-sulfamethoxazole compared to their naïve *E. coli* recipient strains. Interestingly, when compared to the *S. flexneri* donor isolates, the transconjugants and transformants in general had about 2- to 8-fold lower MIC levels for some of the quinolones tested. This result suggests the novel *S. flexneri* isolates may have additional mechanisms to resist quinolones not harbored on the plasmid(s) transferred to the transconjugants and transformants.

### Whole genome analysis of *S. flexneri* strains isolated from macaques

To corroborate antibiotic resistance phenotypes, whole genome sequences of two representative *S. flexneri* isolates, 06-2384 and 06-3102, were obtained (summarized in Table 2 and Supplementary Table 1). The genomes for *S. flexneri* 06-2384 and 06-3102 were assembled into 5 and 8 contigs, respectively. The largest contig for both isolates was ~4.7 Mb with a G+C content of ~50.9%, which is consistent with the chromosomal sequence of other *S. flexneri* genomes (Jin et al., 2002; Wei et al., 2003; Shen et al., 2017). Using PlasmidFinder-1.2, plasmid replicon sequences were detected in the remaining contigs, suggesting they are plasmids. Additionally, the top 5 BLAST hits for these contigs corresponded to plasmids sequences from *Shigella* spp., *E. coli, S. enterica, K. pneumoniae*, and *Citrobacter freundii* (Supplementary Table 2). Thus, *S. flexneri* 06-2384 and 06-3102 strains both appear to harbor several different plasmids in addition to their chromosome. This agrees with Figure 1 in that several bands were apparent after separating plasmid DNA. Despite representing the serotype variants Y and 5b, both 06-2384 and 06-3102 were identified as ST245 by *in silico* MLST and had an ANI of 99.95% and a dDDH of 99.80%, indicating substantial genetic similarity. However, the difference in serotypes as well as the number/size of plasmids (Figure 1 and Table 2) suggests more than one strain variant was present in the NHP colony.

**Table 2:** Genomic characteristics of representative *Shigella flexneri* isolates.

**Isolate**	**Contig No.^a^**	**Contig size (bp)**	**G+C content (%)**	**Protein coding sequences**	**Antibiotic resistance genes^b^**	**Virulence genes^c^**	**Plasmid type^d^**	**GenBank accession**
*S. flexneri* 06-2384	1	4,687,966	50.88	5,092	*aadA1*, *aac(3)-IId, blaOXA-1, oqxA, oqxB, catA, tet(B)*	*ipaH9.8, gad* (2 copies), *sigA, pic, lpfA*	–	NXME00000000
	2	214,255	45.89	314	–	*capU, ipaD, virF*	IncFII	
	3	87,520	48.39	119	–	–	ColRNAI	
	4	60,354	50.64	86	*blaTEM-1B, qnrS1*	–	IncN	
	5	15,063	45.43	17	–	–	ColRNAI	
*S. flexneri* 06-3102	1	4,731,519	50.87	5,182	*aadA1*, *aac(3)-IId, blaOXA-1, oqxA, oqxB, catA, tet(B)*	*ipaH9.8, gad* (2 copies), *sigA, pic, lpfA*	–	NXMF00000000
	2	211,922	44.65	299	–	*capU, ipaD, virF*	IncFII	
	3	86,076	51.83	108	*blaCTX-M-14*	–	IncFII	
	4	6,2689	48.22	81	–	–	IncN	
	5	52001	50.82	75	*blaTEM-1B, qnrS1*	–	ColRNAI	
	6	19,452	45.33	25	–	–	ColRNAI	
	7	12,688	48.84	17	–	–	ColRNAI	
	8	9,913	53.04	23	–	–	ColRNAI	

a Graphical circular maps of the chromosome and plasmid sequences available in Supplementary Figure 3.

b Antibiotic resistance genes detected using ResFinder 2.1.

c Virulence factor genes detected using VirulenceFinder 1.5.

d *Plasmid type predicted using PlasmidFinder 1.3*.

### Antibiotic resistance genes detected by whole genome analysis

ResFinder-2.1 identified several antibiotic resistance genes in the chromosome and plasmid sequences (Table 2). Aminoglycosides (*aadA1, aac(3)-IId*), beta-lactams (*blaOXA-1*), quinolones (*oqxA, oqxB*), chloramphenicol (*catA*), and tetracycline (*tet(B)*) resistance genes were detected in the chromosomes of both strains. In both genomes, *oqxA, oqxB*, and *aac(3)-IId* and *aadA1, blaOXA-1, catA1*, and *tet(B)* clustered together in separate regions. Both of these gene clusters were flanked by several mobile element protein, transposase, and integrase gene annotations. Mutations in the genes for DNA gyrase and topoisomerase subunits A and B were not identified in either of the genomes. Colistin resistance genes (*mcr-1, mcr-2*, and *mcr-3*) were also not identified.

**Figure 2:**
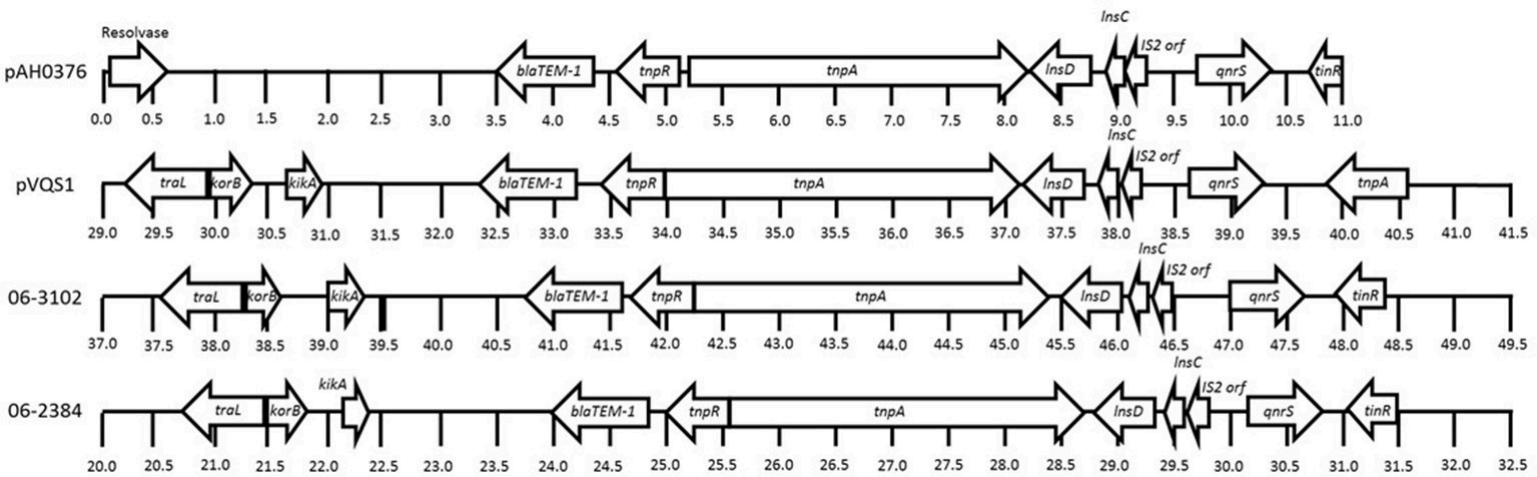
Comparative alignment showing the *blaTEM-1B, qnrS*, and flanking mobile element protein genes from the plasmid sequences of pAH0376 (*Shigella flexneri* 2b; GenBank: AB187515.1), pVQS1 (*Salmonella enterica*; GenBank: JQ609357.1), and *Shigella flexneri* 06-2384 and 06-3102 have nearly identical homology and synteny. Arrowheads represent annotated protein coding sequences and direction of their transcription. Numbers below each sequence represent distance expressed in kilobases.

The *qnrS* gene and beta-lactam resistance gene *blaTEM-1B* were detected on the 60 and 52 kb plasmid contigs for *S. flexneri* 06-2384 and 06-3102, respectively. BLAST analysis found these plasmid contigs were homologous to the completely-sequenced ~40 kb, conjugative plasmid pVQS1 from *S. enterica* (Karczmarczyk et al., 2012) and the partially-sequenced ~47 kb, conjugative plasmid pAH0376 from *Shigella flexneri* 2b (Hata et al., 2005), both of which harbor functional *blaTEM-1B* and *qnrS1* resistance genes. The annotated *qnrS* genes from the novel *S. flexneri* isolates showed 100% sequence identity and coverage to the *qnrS1* genes found in pAH0376 and pVQS1. In general, the resistance genes and flanking mobile element protein genes from *S. flexneri* 06-2384 and 06-3102 showed >98% identity and similar synteny to pVQS1 and pAH0376, except for the transposase genes *tinR* vs. *tnpA*, which had 31%, identify homology (Figure 2). Bidirectional BLASTP analysis using PATRIC's proteome comparison tool indicated >90% of the annotated genes in pVQS1 shared >97% identity to those of the *S. flexneri* 06-2384 and 06-3102 plasmid sequences, including homologs for replication and conjugation genes. This suggests these plasmid sequences from *S. flexneri* 06-2384 and 06-3102 have the potential for self-replication and ability to transfer to new hosts. The *S. flexneri* 06-2384 and 06-3102 plasmid sequences were identical to each other, except 06-3102 appears to contain a duplication of the region encoding *qnrS* and associated mobile element proteins. This second *qnrS* gene is truncated into a predicted 106 amino acid product and is likely non-functional as a result.

*S. flexneri* 06-3102 also contained the beta-lactam resistance gene *blaCTX-M-14* on a ~86 kb plasmid contig. BLAST analysis of this plasmid contig detected >99% identity to the ~74 kb IncFII2 plasmid pAC2901 from *C. freundii* strain AC290 that also encodes the *blaCTX-M-14* resistance gene. The remaining plasmid contig sequences from *S. flexneri* 06-2384 and 06-3102 showed BLAST homology to plasmids from *Enterboacteriaceae* species (Supplemental Table 2). These plasmid sequences were typed as ColRNAI using PlasmidFinder1.2, primarily contained hypothetical proteins, and some encoded phage-related gene annotations. Interestingly, the 15 and 19 kb plasmids from *S. flexneri* 06-2384 and 06-3102, respectively, were enriched for ferric enterobactin uptake protein *FepE* (8/17 and 9/25 annotations for *S. flexneri* 06-2384 and 06-3102, respectively), suggesting a role in iron acquisition.

### Virulence factor genes detected by whole genome analysis

Using VirulenceFinder1.5, virulence factor genes were detected on the chromosome and ~210 kb plasmid sequences from both isolates (Table 2). Additional potential virulence factor genes were also identified by BLASTP from the VFDB, Victors, and PATRIC_VF databases using the specialty genes feature hosted by PATRIC. This included 998 and 1,009 virulence factor genes detected in *S. flexneri* 06-2384 and 06-3102, respectively, with functions ranging from host adherence and invasion to biofilm formation and iron acquisition (Supplemental Table 3).

Of particular note, several serine protease autotransporters of *Enterobacteriaceae* (SPATE) enterotoxin genes were identified, which are implicated in diarrheal illness and gastrointestinal cytotoxicity by pathogenic *Shigella* spp. and *E. coli* infection (Dautin, 2010; Mattock and Blocker, 2017). For both *S. flexneri* 06-2384 and 06-3102, these include the chromosomally-encoded *Shigella* enterotoxin 1 (*ShET1*), protease involved in intestinal colonization (*Pic*), and *Shigella* extracellular protein A (*SigA*) genes, which have been shown to exert cytotoxicity to epithelial cells, mediate serum resistance, induce fluid secretion, and degrade mucus/mucin (Dautin, 2010; Faherty et al., 2012; Mattock and Blocker, 2017). Two paralogs for *ShET2* along with type III secretion system (T3SS) components and conjugative transfer genes were identified on the ~210 kb, IncFII plasmids in both *S. flexneri* 06-2384 and 06-3102. These *ShET2* paralogs are T3SS effector proteins that induce secretory activity by the gastrointestinal epithelium to cause fluid-laden diarrhea (Farfan et al., 2011; Faherty et al., 2012; Mattock and Blocker, 2017).

## Discussion

In this study, five novel, multiple-antibiotic resistant *S. flexneri* isolates were cultured from the feces of three macaques maintained in a biomedical research colony. MIC testing demonstrated these *S. flexneri* isolates were resistant or have decreased susceptibility to beta-lactam, cephalosporin, aminoglycoside, tetracycline, and quinolone, but were susceptible to macrolide and sulfonamide antibiotic classes. Treatment with trimethoprim-sulfadiazine successfully eradicated *S. flexneri* infection in two of these animals, while infection in the third appeared to be self-limiting after post-surgical use of enrofloxacin. The purpose of this study was to understand the potential mechanisms of multi-drug resistance in these novel *S. flexneri* isolates.

PCR of plasmid-enriched DNA detected the *qnrS*, but not *qnrA* or *qnrB*, in all of the *S. flexneri* isolates. The sequences of the *qnrS* genes had 100% homology to the plasmid-encoded *qnrS1* genes from *S. flexneri* and other *Enterobacteriaceae* species with quinolone resistance phenotypes. This suggested quinolone resistance in the novel *S. flexneri* isolates was mediated by a plasmid-encoded *qnrS1* gene. To test this, quinolone-susceptible *E. coli* strains were introduced with plasmid DNA from the novel *S. flexneri* isolates via conjugation or transformation and then tested for antibiotic resistance. Plasmid DNA isolated from the transconjugants and transformants was PCR-positive for the *qnrS* gene. The transconjugants and transformants demonstrated decreased susceptibility to quinolones and resistance to beta-lactam antibiotics compared to their respective naïve *E. coli* recipient strains. When compared to their respective *S. flexneri* donor isolates, the transconjugants and transformants in general had lower resistance to the quinolones.

To further support that the novel *S. flexneri* isolates harbored plasmid-encoded *qnrS* genes and to identify other mechanisms for quinolone resistance, whole genome sequences were obtained for two representative isolates. Consistent with the MIC results, antibiotic resistance genes for aminoglycosides (*aadA1, aac(3)-IId*), beta-lactams (*blaOXA-1*), quinolones (*oqxA, oqxB*), and tetracycline (*tet(B)*) were detected in their chromosomes. The *catA* was also detected in the chromosomes, suggesting these isolates have the potential for chloramphenicol resistance, although this was not experimentally confirmed. The *oqxA* and *oqxB* genes have been previously identified in *E. coli, K. pneumoniae*, and *K. oxytoca* isolates and encode multi-drug efflux pumps against quinolones that yield low-level resistance (Poirel et al., 2012; Andres et al., 2013; Rodriguez-Martinez et al., 2013).

In both *S. flexneri* genomes, *qnrS* was co-associated with *blaTEM-1B* on plasmid-like sequences that had identical homology and synteny to pAH0376 and pVQS1 from *S. flexneri* 2b and *S. enterica*, respectively. Additionally, as observed for pAH0376 and pVQS1, these plasmids could be experimentally transferred to *E. coli*, conferring the recipients with quinolone and beta-lactam resistance (Hata et al., 2005; Karczmarczyk et al., 2012). The activity of *oqxA* and *oqxB* combined with *qnrS* may enhance overall quinolone resistance in these *S. flexneri* isolates and may explain why the *E. coli* recipients demonstrated less resistance to quinolones compared to their donor counterparts.

A recently published study surveyed the prevalence of antimicrobial resistance in zoonotic pathogens cultured from NHPs across several different biomedical research institutions in the United States (Kim et al., 2017). The authors found that enrofloxacin is the primary antibiotic chosen by veterinarians for treating diarrhea and gingivitis caused by presumptive *S. flexneri* infection, and while diagnostic labs commonly screen *S. flexneri* isolates for enrofloxacin resistance, no resistant strains were reported. A major caveat of this study is the small number of participating institutions; only seven institutions participated, and antibiotic resistance data was obtained from just three. Likewise, the methods used to detect and characterize antibiotic resistance potential were inconsistent between institutions. Therefore, estimations of resistance to enrofloxacin and other quinolones by *S. flexneri* could have been underreported or missed.

The World Health Organization (WHO) classifies quinolones as the highest priority of clinically important antibiotics based on it being a limited available therapy option to treat serious bacterial infections with a high risk of zoonosis and an ability to acquire antibiotic resistance genes (WHO, 2017). The Centers for Disease Control and Prevention (CDC) recognizes that *Shigella* spp. are becoming increasingly resistant to fluoroquinolones and other antibiotics (CDC, 2017). The CDC recommends that fluoroquinolones should be avoided when the MIC for ciprofloxacin is ≥0.12 μg/mL, even though this is classified as “susceptible” according to CLSI criteria. The novel *S. flexneri* isolates and their *E. coli* transconjugants/transformants in this study had MICs for ciprofloxacin of 0.25–2 μg/mL, which though below the CLSI criteria for resistance, meet the CDC's standard for excluding fluoroquinolones to treat *Shigella* spp.

According to the latest report by the National Antimicrobial Resistance Monitoring System (2016), as well as the survey by Kim et al. (2017), *S. flexneri* isolates from humans and NHP's frequently demonstrate resistance to more than one class of antibiotics. *Shigella* spp. can also exhibit multi-drug resistance with substantial diversity observed in their resistance profiles. As observed for the *S. flexneri* isolates in this study, resistance or decreased susceptibility to beta-lactam, cephalosporin, aminoglycoside, and tetracycline antibiotic classes, along with quinolones, was noted. The co-association of *qnrS1* and *blaTEM-1B* and other antibiotic resistance gene clusters poses a particular risk in predicting successful eradication strategies. Therefore, culture for *Shigella* spp. and other enteric pathogens followed by antibiotic testing should be perform prior to initiation of treatment.

The finding of quinolone-resistant *S. flexneri* in this study may reflect the common use of enrofloxacin to preemptively treat suspected shigella-induced diarrheal disease commonly encountered in newly imported macaques from Asia (Fox, 1975; Tribe and Fleming, 1983; Line et al., 1992; Banish et al., 1993). Macaques co-housed or maintained in groups can also readily acquire *Shigella* spp. infections from other infected monkeys via fecal/oral or oral/oral routes (Shipley et al., 2010). Cross-transmission of *Shigella* spp. could have occurred in our colony because animals were co-housed in adjacent cages within the same animal facility. In two of the three rhesus monkeys, a standard enrofloxacin regimen failed to eradicate the *S. flexneri* infection, but trimethoprim-sulfadiazine treatment was successful. Trimethoprim-sulfamethoxazole has been reported to successfully eradicate *Shigella* spp. infection in a colony of wild-caught rhesus monkeys (Pucak et al., 1977). *S. flexneri* in the third rhesus was eliminated during a 10-day course of enrofloxacin treatment. Shigellosis in humans is known to typically resolve within 5–7 days without antibiotic treatment (Hale and Keusch, 1996), so it is possible infection was self-limiting in this individual.

Humans can become infected with *Shigella* spp. through contact with NHPs in research or zoo setting or maintained as pets (Fox, 1975; Tribe and Fleming, 1983; Kennedy et al., 1993). *Shigella* spp. infection in humans can readily transmit from person-to-person or through contaminated food or water (Scallan et al., 2011). Additionally, ciprofloxacin has continued to be one of the most commonly prescribed antibiotics for treating shigellosis in human patients despite the discovery of ciprofloxacin-resistant *Shigella* spp. and plasmid-mediated quinolone resistant strains of *S. flexneri* (Bowen et al., 2015; CDC, 2017). Therefore, *S. flexneri* isolates with the capacity to spread and acquire quinolone resistance genes represent not only an institutional concern when treating NHPs, but also a public health concern considering the zoonotic potential of these pathogens. Strict hygienic principles should also be implemented and always followed by personnel in contact with NHPs in order to reduce risk of acquiring infection.

In conclusion, this study demonstrates for the first time the discovery of plasmid-mediated quinolone resistance genes in multiple-antibiotic drug resistant *S. flexneri* isolated from research macaques. Given that antibiotic treatments, such as enrofloxacin, are often prescribed empirically and prophylactically, the findings of this study stress the need to test for antibiotic susceptibility in NHPs with suspected *Shigella* spp. infection beforehand in order to appropriately treat these animals and reduce zoonotic risk.

## Ethics statement

All procedures were performed under animal protocols approved by the Massachusetts Institute of Technology Committee on Animal Care (CAC) and in accordance with the guidelines for animal care at Massachusetts Institute of Technology. Animals were cared for in an AAALAC International-accredited animal facility at MIT under federal, state, local and NIH guidelines for animal care.

## Author contributions

AM, HM, ZS, JD-F, and AG characterized novel Shigella isolates. EB cultured novel Shigella isolates. HM, RM, and MP provided veterinary clinical work up of non-human primates, including sample collection. AM, HM, JD-F, and JF wrote and/or reviewed manuscript.

### Conflict of interest statement

The authors declare that the research was conducted in the absence of any commercial or financial relationships that could be construed as a potential conflict of interest.
