# Supplementary figures and images for: Plasmid-Mediated Quinolone Resistance in Shigella flexneri Isolated From Macaques
Source: Front Microbiol. 2018 Mar 5;9:311. doi: 10.3389/fmicb.2018.00311 (PMC5844971; doi:10.3389/fmicb.2018.00311)

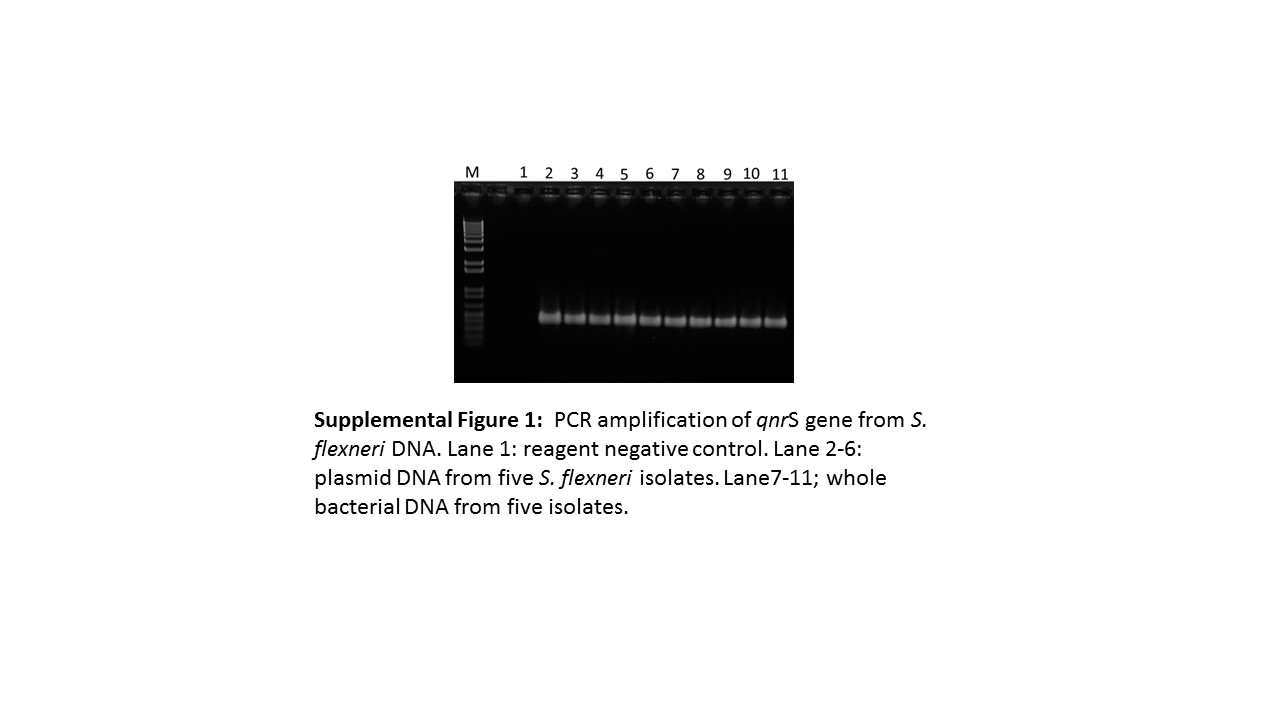

Supplement: Supplementary file 1 [file Image1.JPEG]

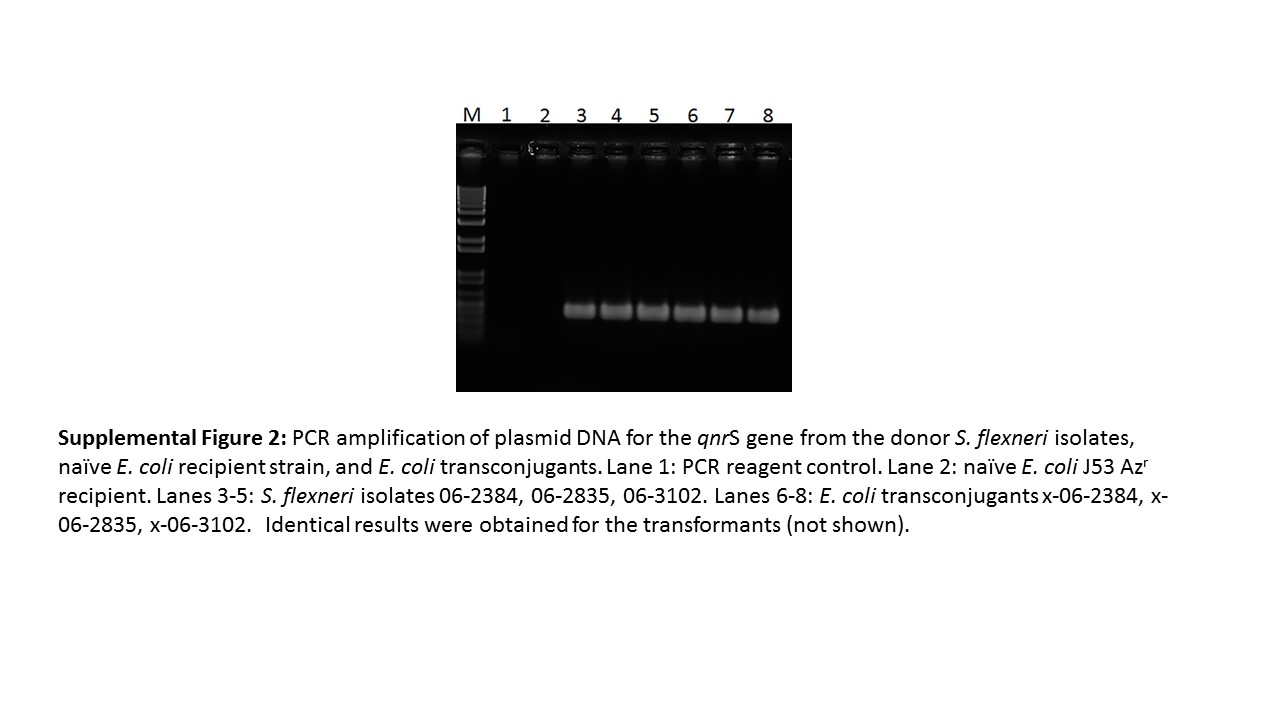

Supplement: Supplementary file 2 [file Image2.JPEG]
