# Supplementary material for: Plasmid-Mediated Quinolone Resistance in Shigella flexneri Isolated From Macaques
Source: Front Microbiol. 2018 Mar 5;9:311. doi: 10.3389/fmicb.2018.00311 (PMC5844971; doi:10.3389/fmicb.2018.00311)
Supplement: Supplementary file 3 [file Image3.PDF]

Supplemental Figure 3: Graphical circular maps of the chromosome and plasmid contigs showing position and orientation of protein coding sequences (CDS), RNA genes, repeat regions, and GC content.

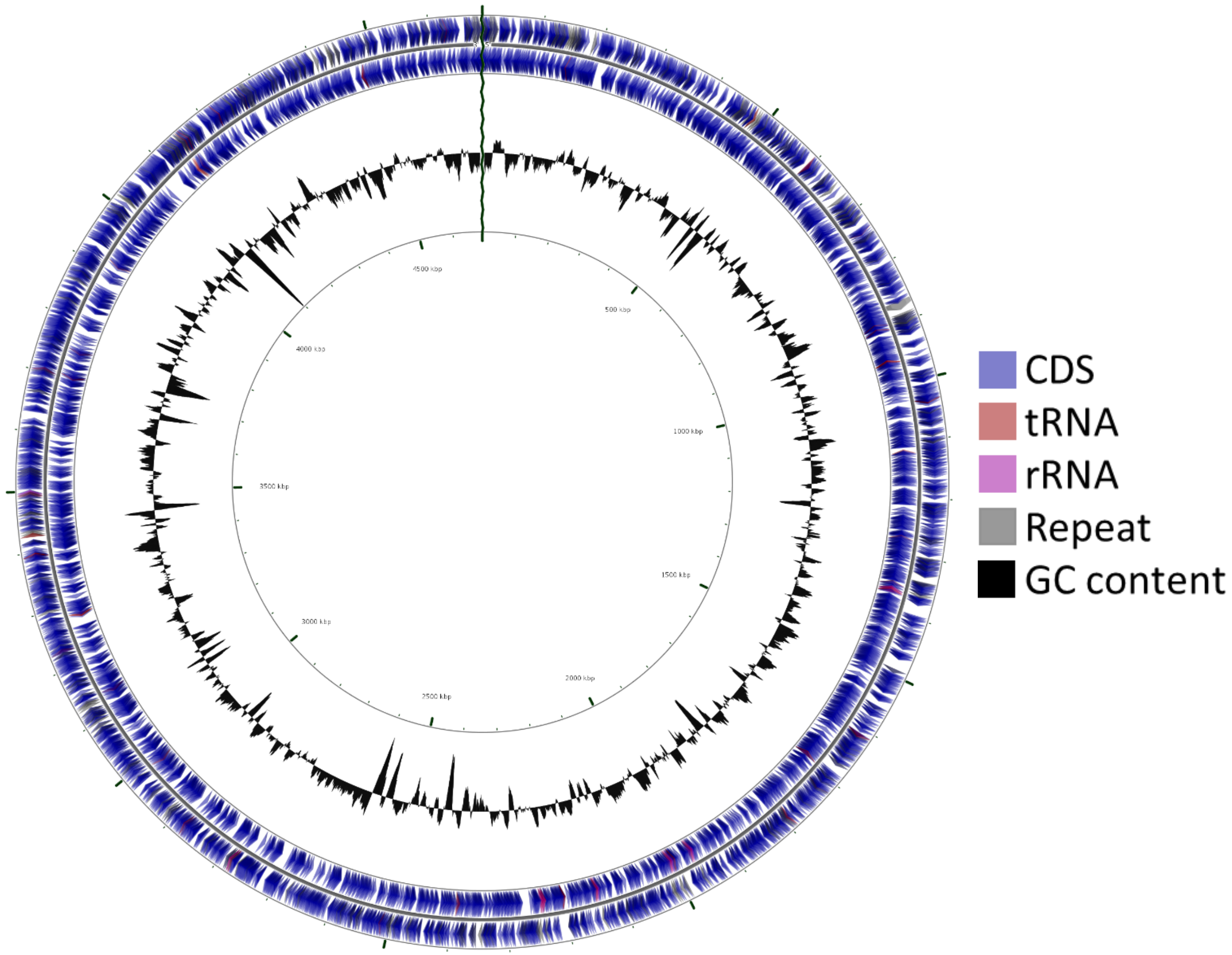

Shigella flexneri strain 06-2384
Contig 1

Supplemental Figure 3: Graphical circular maps of the chromosome and plasmid contigs showing position and orientation of protein coding sequences (CDS), RNA genes, repeat regions, and GC content.

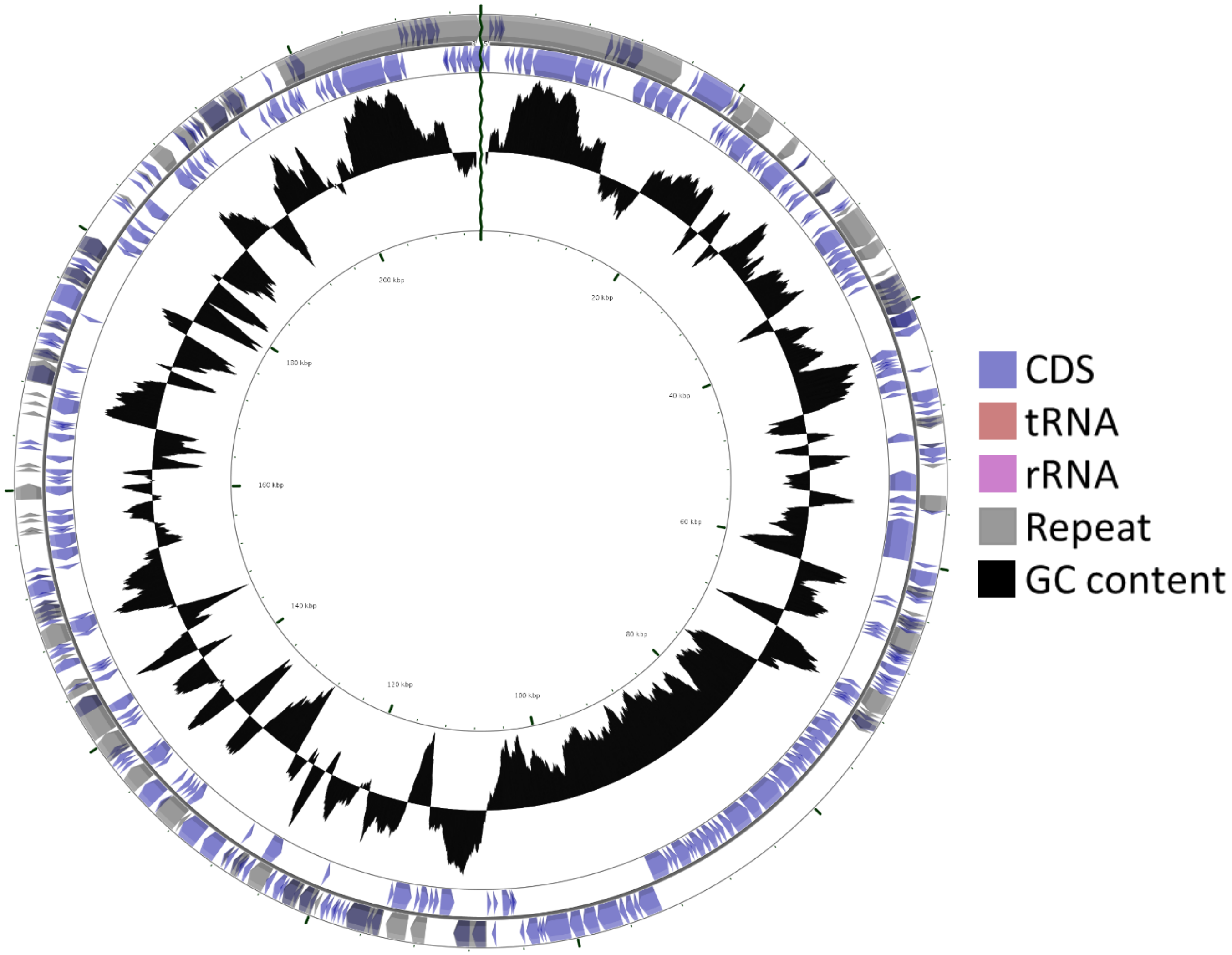

Shigella flexneri strain 06-2384
Contig 2

Supplemental Figure 3: Graphical circular maps of the chromosome and plasmid contigs showing position and orientation of protein coding sequences (CDS), RNA genes, repeat regions, and GC content.

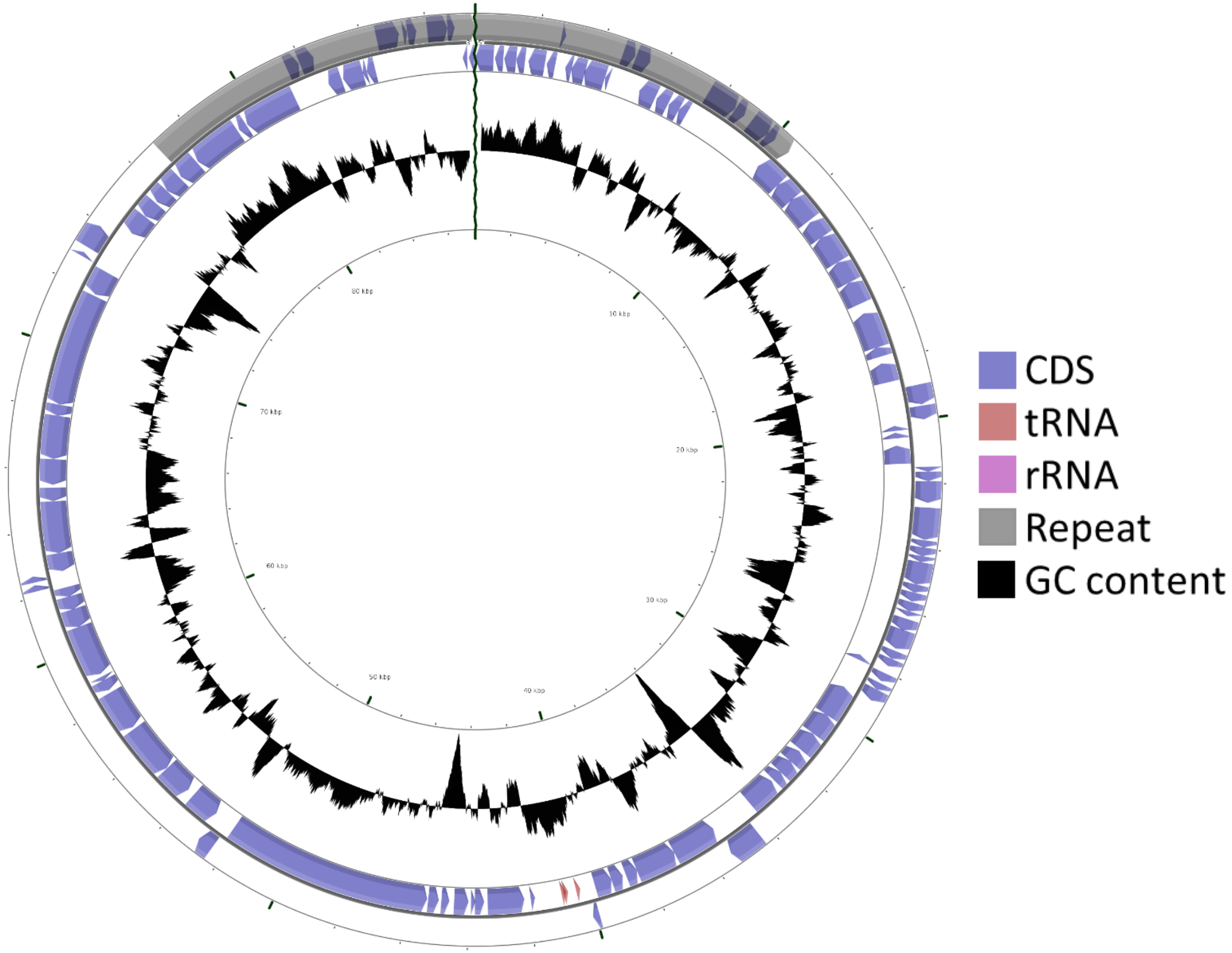

Shigella flexneri strain 06-2384
Contig 3

Supplemental Figure 3: Graphical circular maps of the chromosome and plasmid contigs showing position and orientation of protein coding sequences (CDS), RNA genes, repeat regions, and GC content.

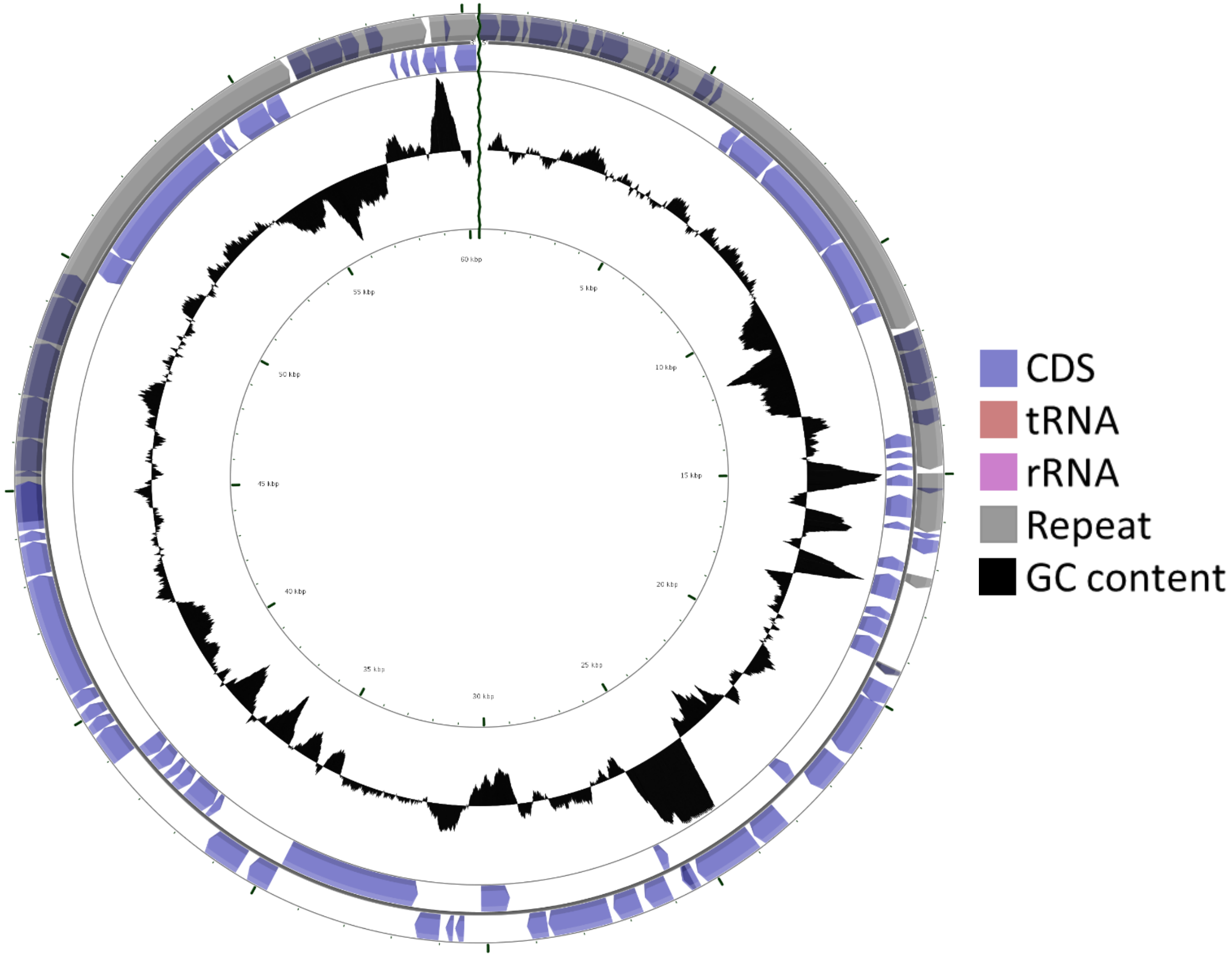

Shigella flexneri strain 06-2384
Contig 4

Supplemental Figure 3: Graphical circular maps of the chromosome and plasmid contigs showing position and orientation of protein coding sequences (CDS), RNA genes, repeat regions, and GC content.

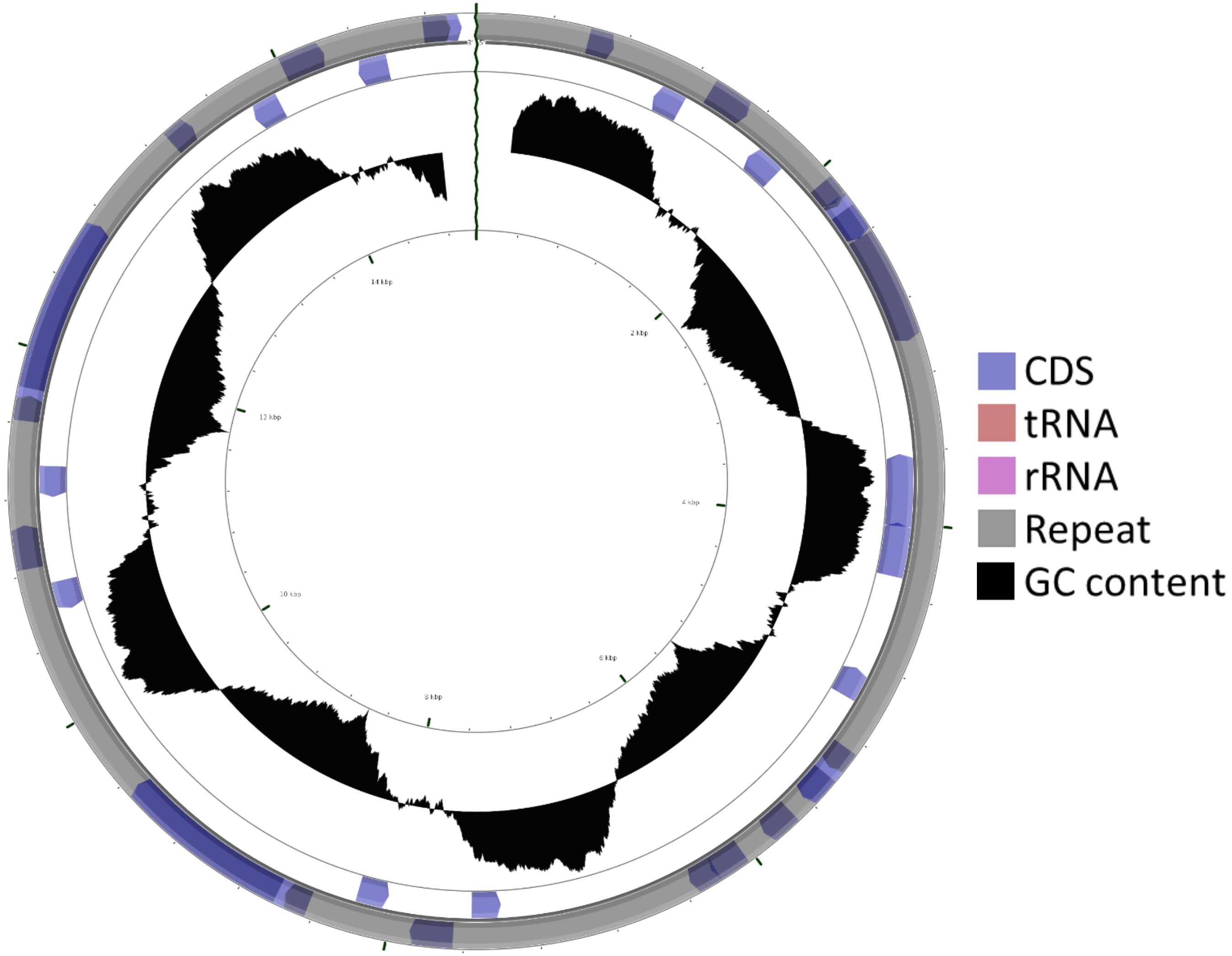

Shigella flexneri strain 06-2384
Contig 5

Supplemental Figure 3: Graphical circular maps of the chromosome and plasmid contigs showing position and orientation of protein coding sequences (CDS), RNA genes, repeat regions, and GC content.

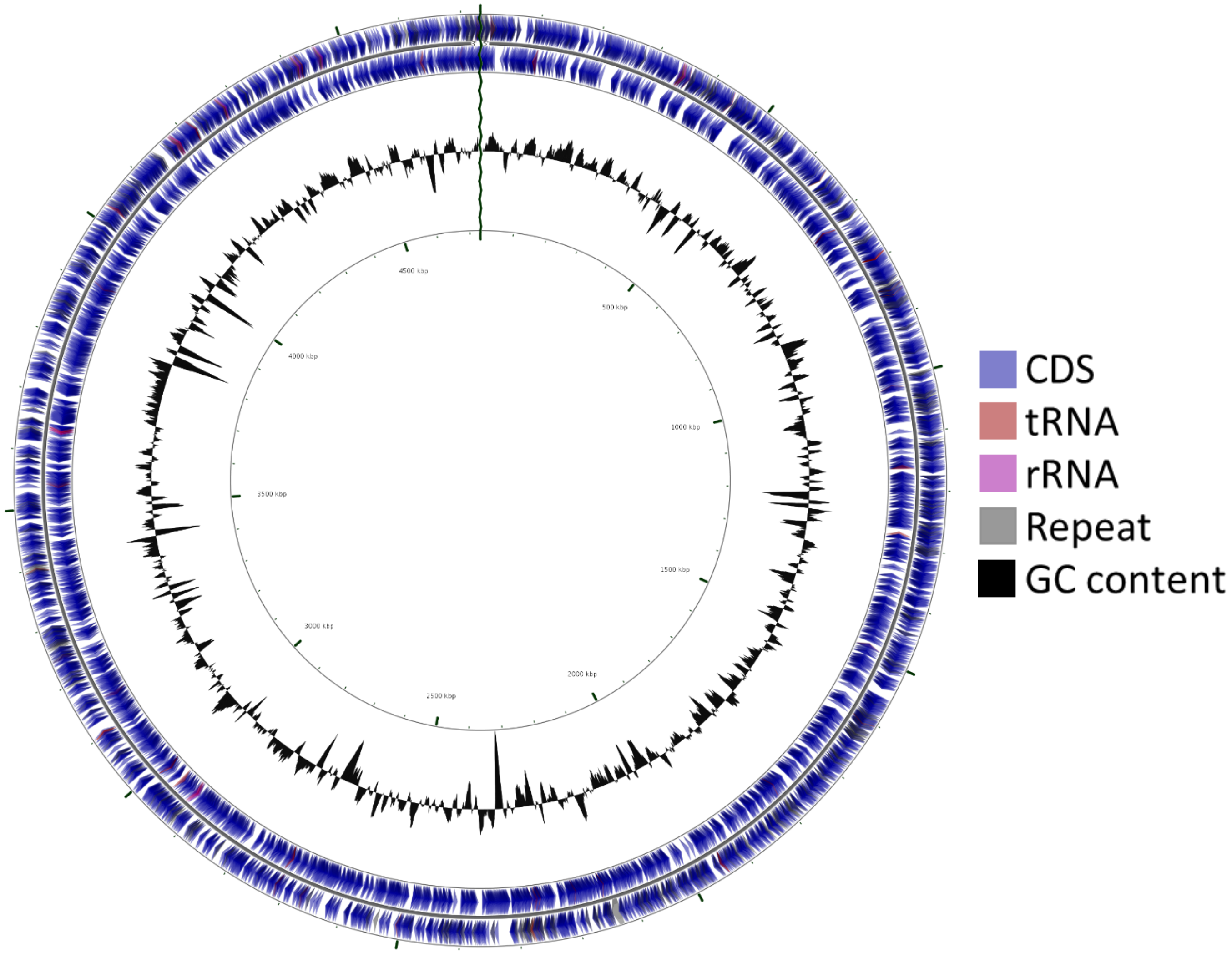

Shigella flexneri strain 06-3102
Contig 1

Supplemental Figure 3: Graphical circular maps of the chromosome and plasmid contigs showing position and orientation of protein coding sequences (CDS), RNA genes, repeat regions, and GC content.

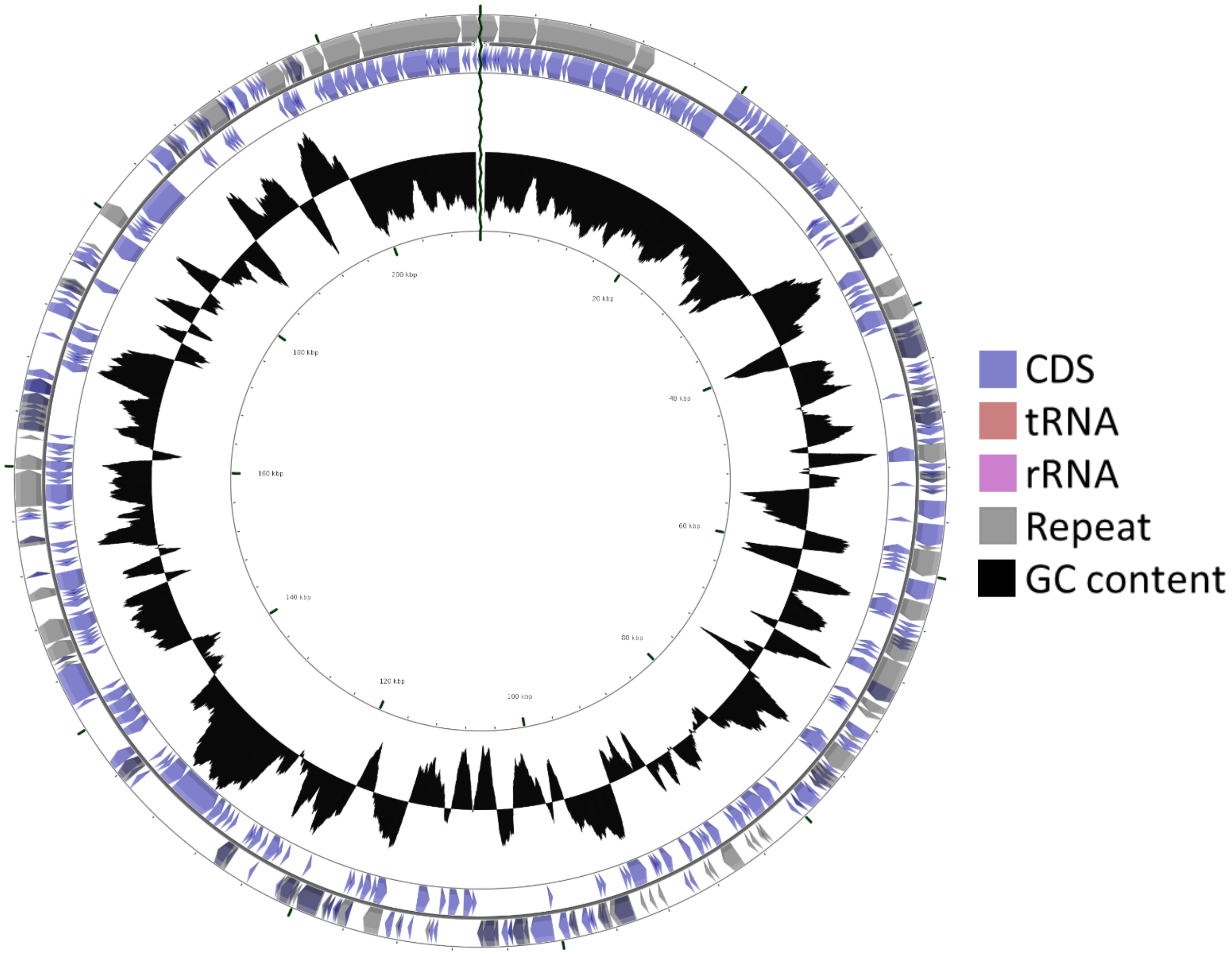

Shigella flexneri strain 06-3102
Contig 2

Supplemental Figure 3: Graphical circular maps of the chromosome and plasmid contigs showing position and orientation of protein coding sequences (CDS), RNA genes, repeat regions, and GC content.

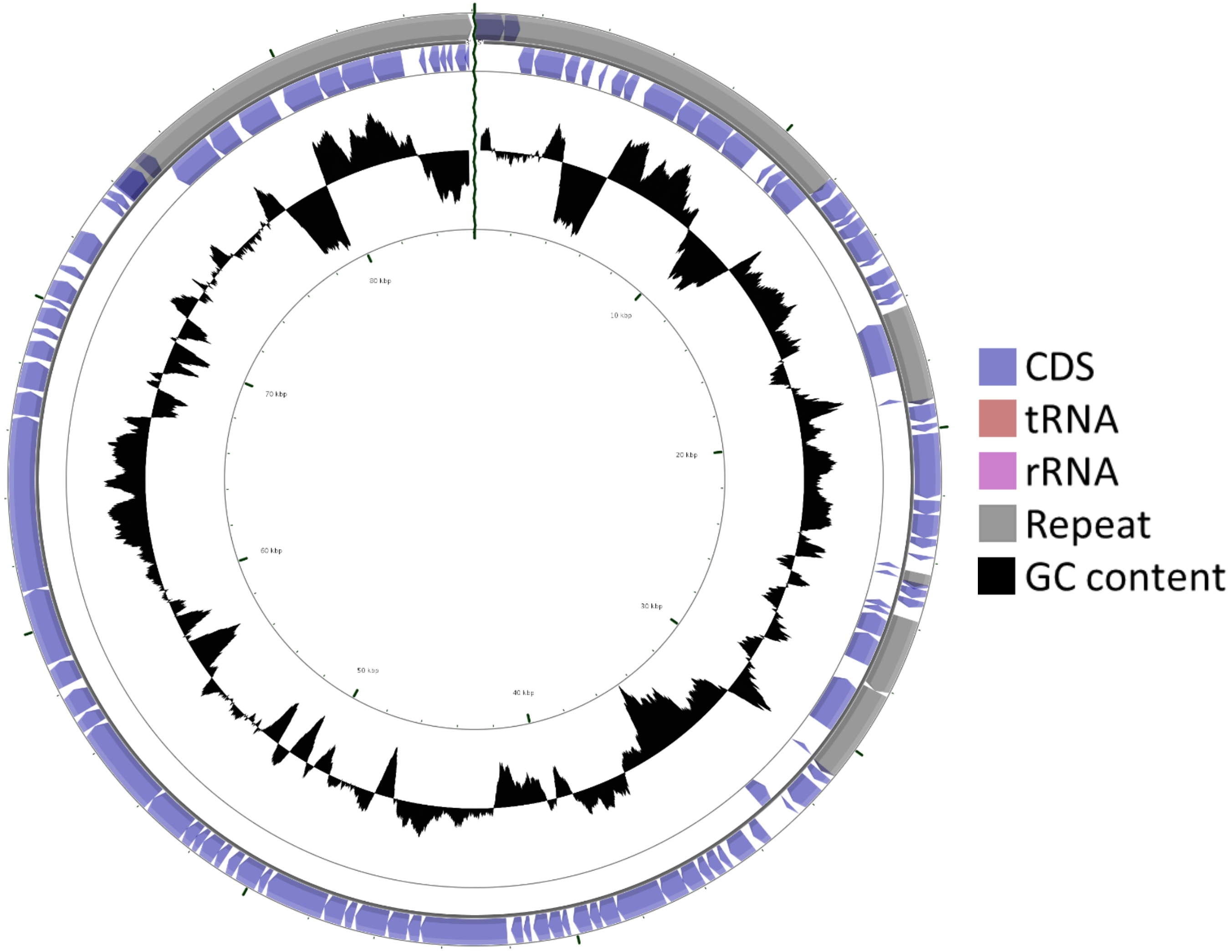

Shigella flexneri strain 06-3102
Contig 3

Supplemental Figure 3: Graphical circular maps of the chromosome and plasmid contigs showing position and orientation of protein coding sequences (CDS), RNA genes, repeat regions, and GC content.

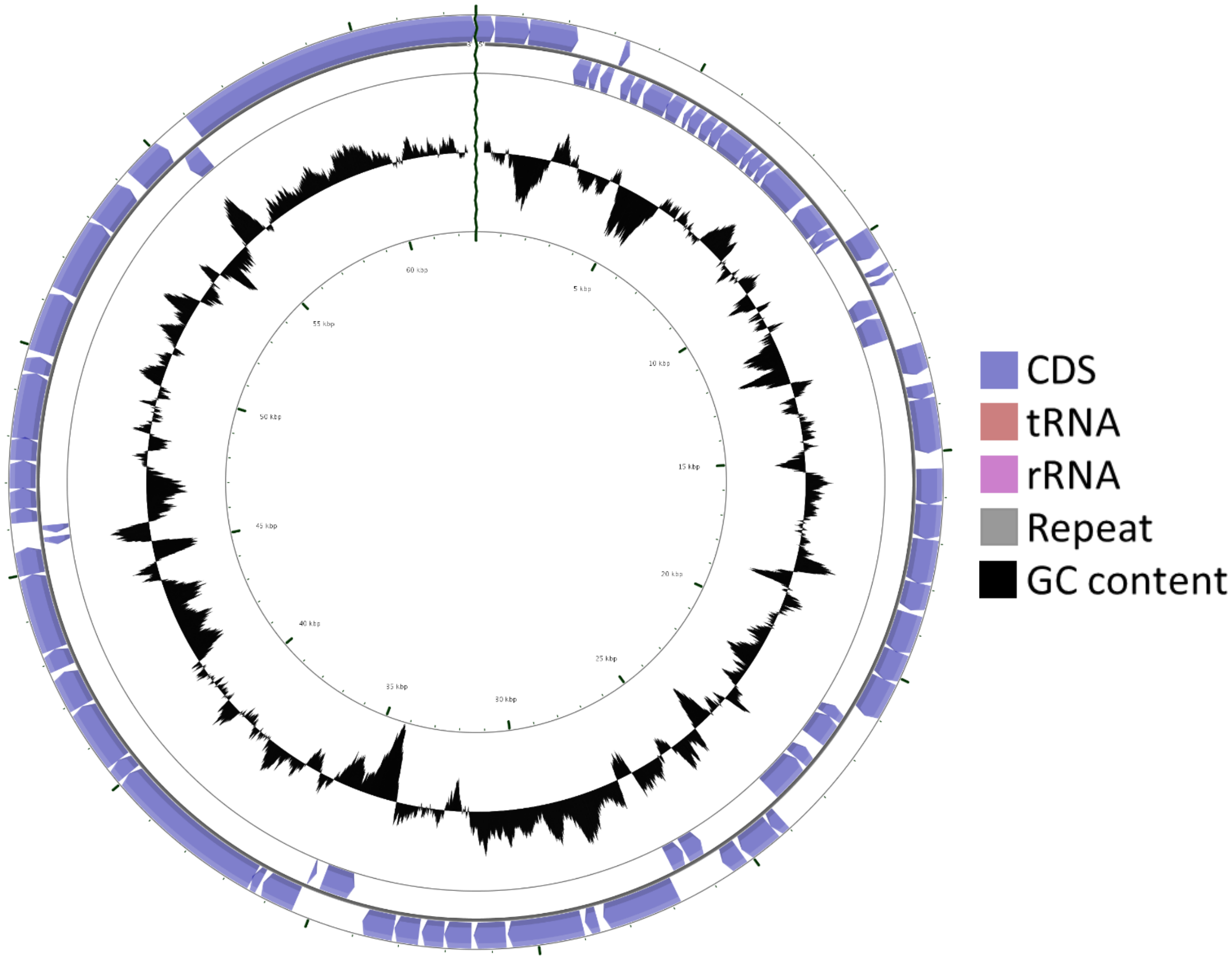

Shigella flexneri strain 06-3102
Contig 4

Supplemental Figure 3: Graphical circular maps of the chromosome and plasmid contigs showing position and orientation of protein coding sequences (CDS), RNA genes, repeat regions, and GC content.

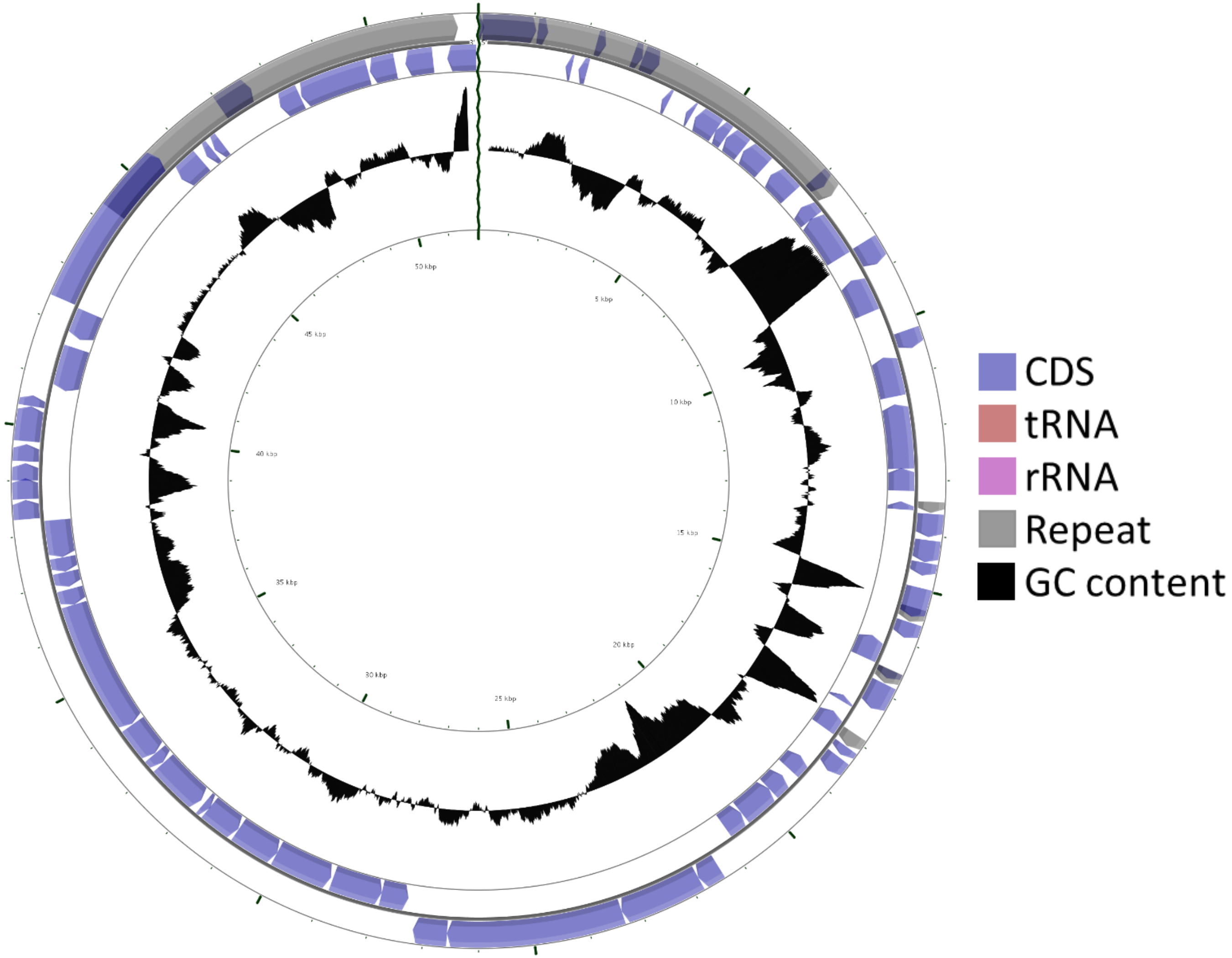

Shigella flexneri strain 06-3102
Contig 5

Supplemental Figure 3: Graphical circular maps of the chromosome and plasmid contigs showing position and orientation of protein coding sequences (CDS), RNA genes, repeat regions, and GC content.

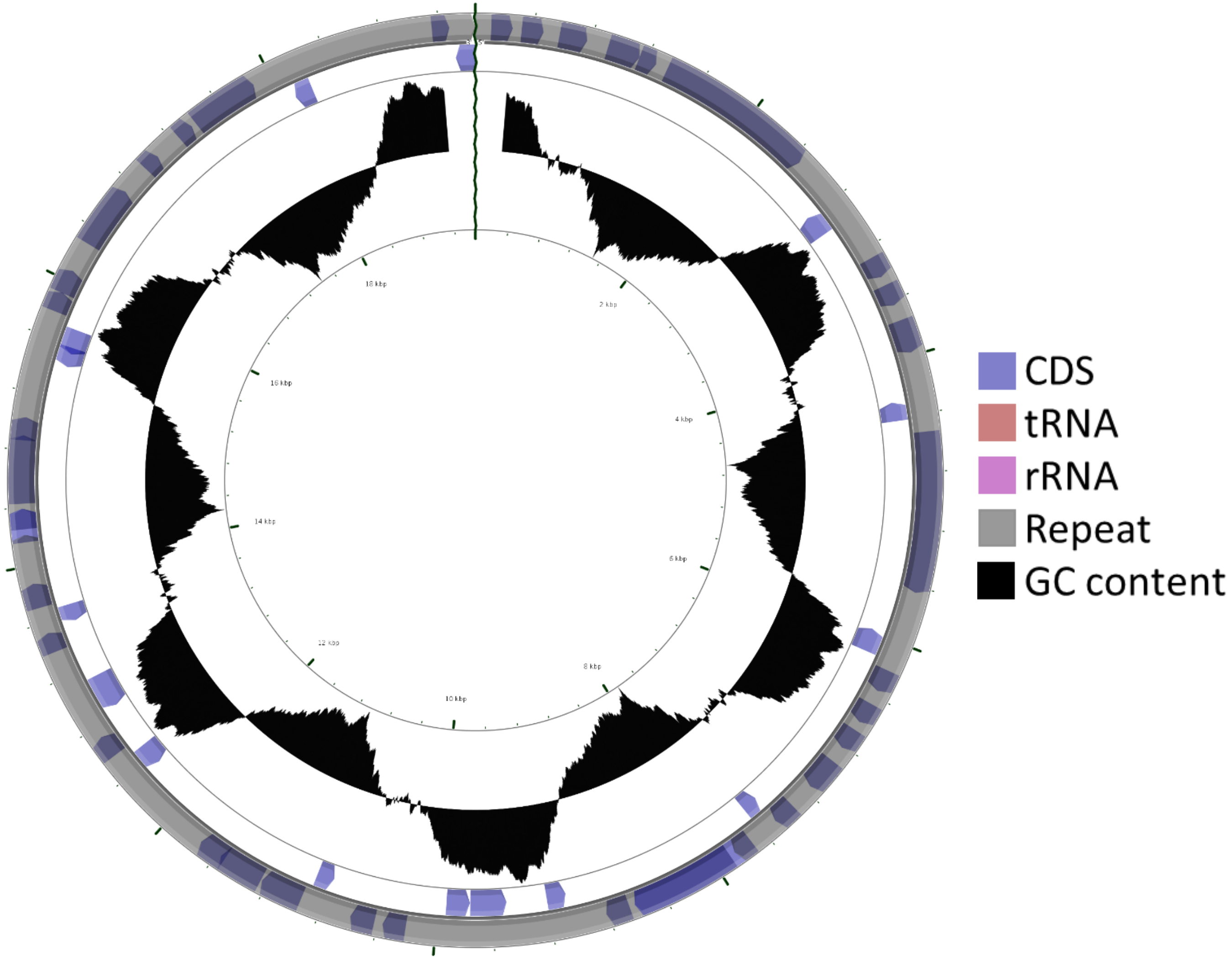

Shigella flexneri strain 06-3102
Contig 6

Supplemental Figure 3: Graphical circular maps of the chromosome and plasmid contigs showing position and orientation of protein coding sequences (CDS), RNA genes, repeat regions, and GC content.

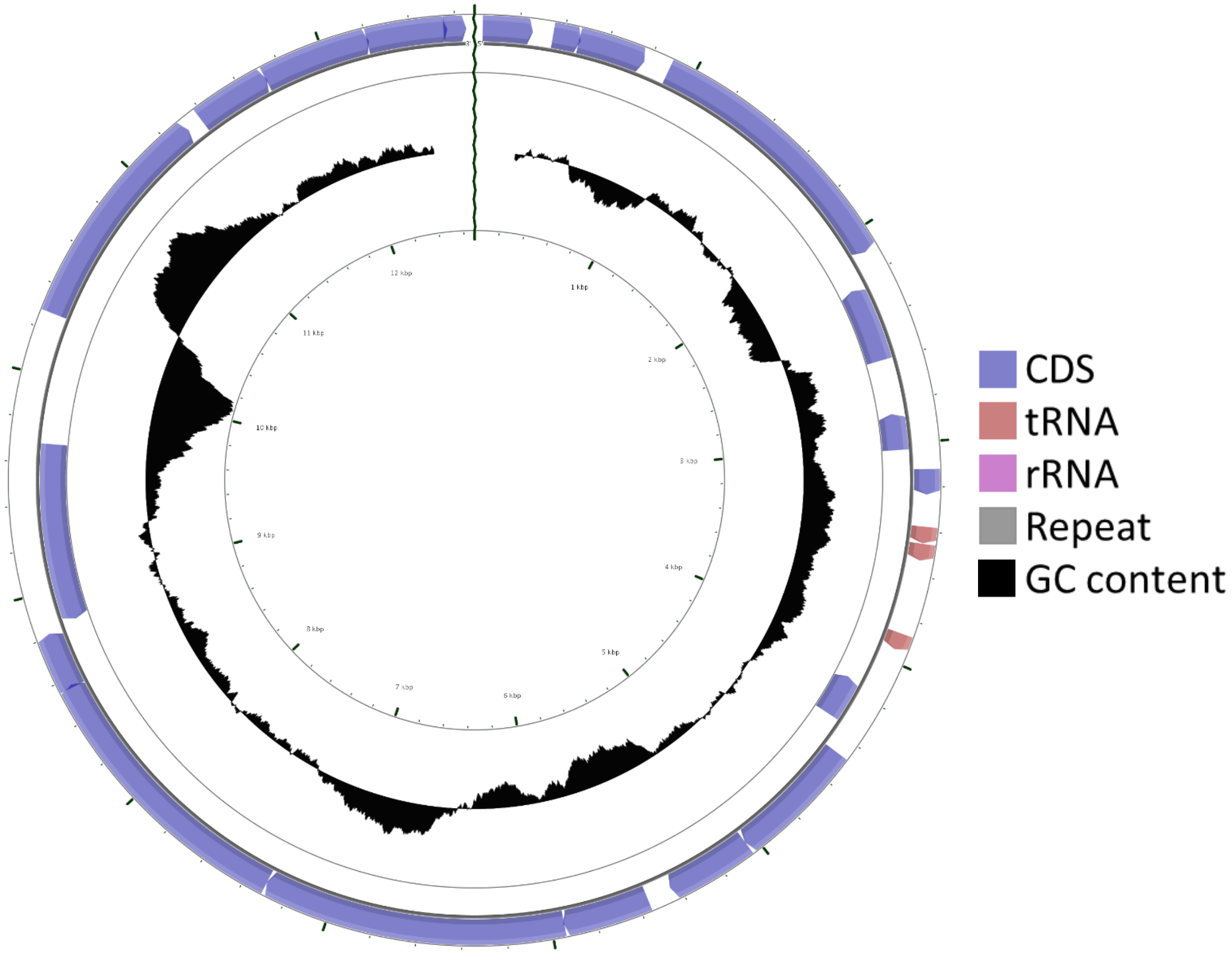

Shigella flexneri strain 06-3102
Contig 7

Supplemental Figure 3: Graphical circular maps of the chromosome and plasmid contigs showing position and orientation of protein coding sequences (CDS), RNA genes, repeat regions, and GC content.

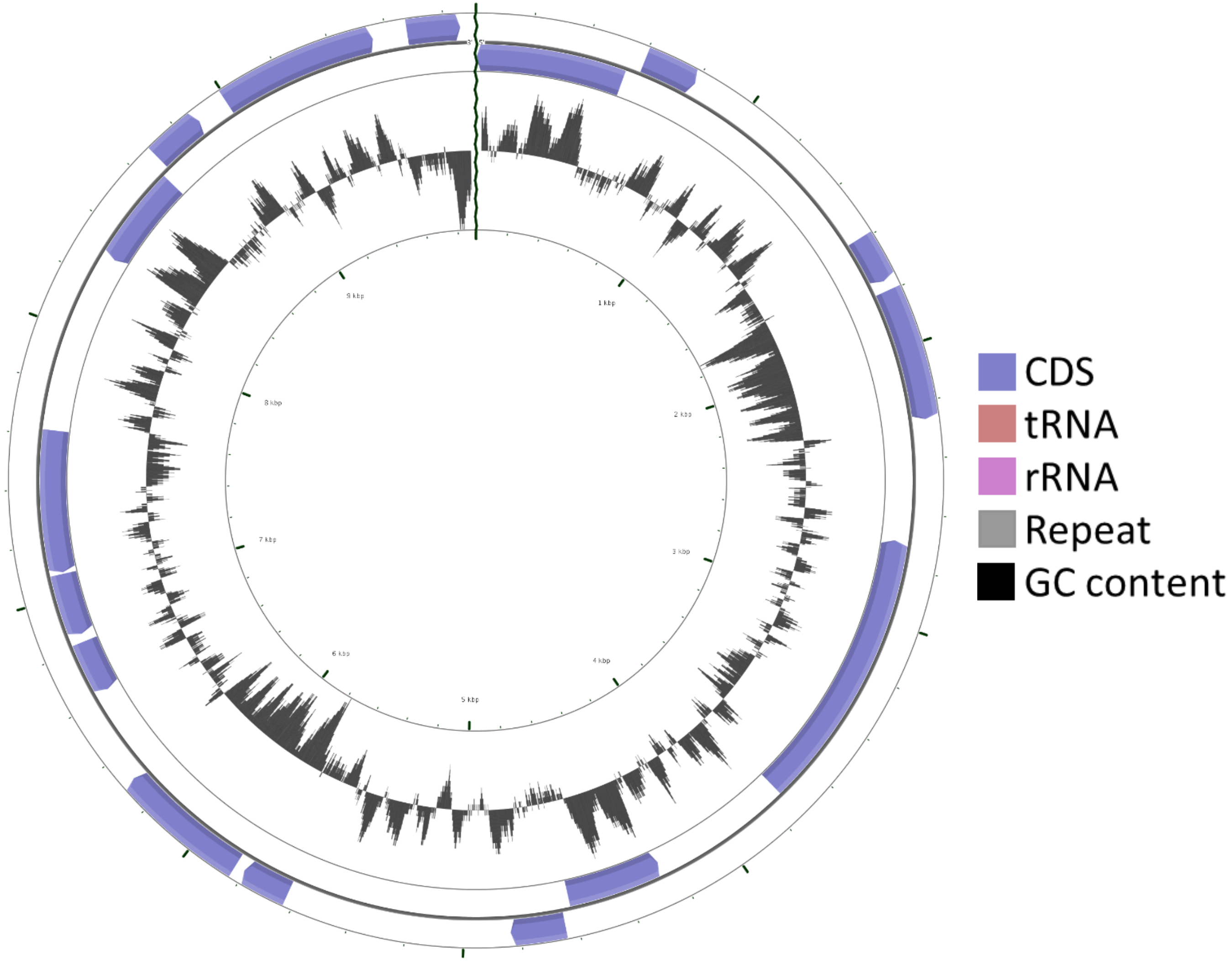

Shigella flexneri strain 06-3102
Contig 8
